# Cytoreductive Surgery and Hyperthermic Intraperitoneal Chemotherapy for Peritoneal Surface Malignancies: Learning Curve Based on Surgical and Oncological Outcomes

**DOI:** 10.3390/cancers12092387

**Published:** 2020-08-23

**Authors:** Jerzy Mielko, Karol Rawicz-Pruszyński, Katarzyna Sędłak, Katarzyna Gęca, Magdalena Kwietniewska, Wojciech P. Polkowski

**Affiliations:** Department of Surgical Oncology, Medical University of Lublin, Radziwiłłowska 13 St., 20-080 Lublin, Poland; jerzy.mielko@umlub.pl (J.M.); sedlak.katarz@gmail.com (K.S.); kasiaa.geca@gmail.com (K.G.); mag.cislo@gmail.com (M.K.); wojciech.polkowski@umlub.pl (W.P.)

**Keywords:** CRS + HIPEC, peritoneal surface malignancies, learning curve

## 1. Introduction

Cytoreductive surgery (CRS) with hyperthermic intraperitoneal chemotherapy (HIPEC) is a complex, highly specialized procedure used to treat peritoneal surface malignancies (PSM) [1,2,3,4]. The PSM includes both rare diseases of the peritoneum (pseudomyxoma peritonei, peritoneal mesothelioma) as well as metastases from various primary cancers (ovarian, colorectal, gastric, etc.) to the surface of peritoneum. Despite initial skepticism, CRS + HIPEC has become a widely accepted procedure in the treatment of pseudomyxoma peritonei, appendiceal cancer, metastatic colorectal cancer, and peritoneal mesothelioma [5,6,7,8,9,10]. Significant improvement in oncological results has also been reported in peritoneal dissemination from gastric and ovarian cancers compared to palliative treatment [11,12]. Achieving complete cytoreduction often requires extensive surgical procedure with multiple anastomoses, associated with relatively high complication rates (14–70%) [13]. In reference centers, perioperative mortality reaches 8%. This high rate has been attributed to the learning curve (LC), and it decreases after about 100–140 operations [14,15,16,17,18]. In the CRS with HIPEC, as in the case of other extensive oncological operations (pancreatoduodenectomy, hemihepatectomy, esophagectomy), lower complication rates were found in high-volume surgical centers [19,20]. The LC evaluation based on the completeness of cytoreduction, morbidity and mortality, prognosis and quality of life can be applicable for both individual surgeons and PSM institutions [17].

The sequential probability ratio test (SPRT) and its modification—the risk-adjusted sequential probability ratio test (RA-SPRT)—was used during World War II and was originally developed as a method of controlling the quality of military supplies. In medical practice, SPRT is one of the statistical tests used to monitor the safety of medical interventions [21]. In the LC assessment, SPRT was carried out in several studies [14,15,17,22,23,24,25,26]. The results were encouraging for institutions willing to introduce HIPEC procedures in strictly selected patients. Precise and critical qualifications for CRS + HIPEC should be considered as an important element of LC. The comprehensive complication index (CCI) is a metric based on Clavien–Dindo classification, which records complications in surgery weighted by severity [27,28]. It summarizes all postoperative complications and is more sensitive than existing morbidity endpoints [27]. Additionally, it allows for the quantification and comparison of multiple complications burden in patients undergoing extensive cytoreduction surgery. To the best of our knowledge, this is the first study that aimed to use SPRT to determine the LC for CRS + HIPEC and assess the minimum number of performed procedures to achieve the necessary experience in the treatment of PSM based on Clavien–Dindo and CCI classifications.

## 2. Results

Detailed demographic data and clinical features of patients treated with CRS + HIPEC are presented in Table 1. 

To achieve complete cytoreduction (CCR0), extensive multi-visceral resections were performed in 99 (48,7%) cases. In 174 (88%) cases, complete cytoreduction (CCR0/1) was performed, while incomplete cytoreduction (CCR2/3) was only performed in only 26 (12%) cases.

From 2010 to 2015, HIPEC was performed using the open (Coliseum) technique in 107 (53%) patients. Between 2016 and 2018, HIPEC was applied with closed technique in 93 (47%) patients, including 3 (1.6%) patients operated with laparoscopic HIPEC. For HIPEC perfusion, mitomycin C was used in 87 patients (43%), whereas oxaliplatin in 116 patients (57%). 

The 30- and 90-day postoperative mortality was 4 (2%) and 8 (4%), respectively. Twenty-two (11%) patients required relaparotomy. Severe surgical complications (Clavien–Dindo grade III or IV) occurred in 47 patients (23%). The mean comprehensive complication index (CCI) score was 31 (Me – 21). The median overall survival (OS) was 45 months in the whole group of patients. One-, 3- and 5-year OS rates were 90%, 60%, and 41%, respectively. 

Characteristics of CRS + HIPEC procedures are presented in Table 2. 

The spectrum of CRS + HIPEC procedures performed in the study is presented in Table 3.

The early results of surgical treatment are summarized in Table 4.

**Figure 1:**
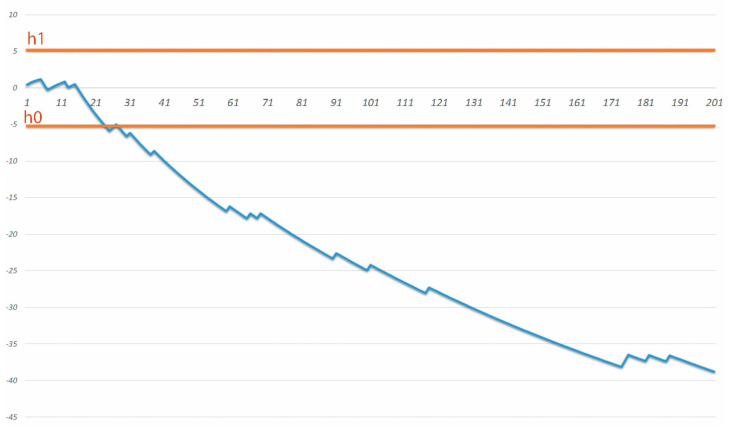
Risk-adjusted sequential probability ratio test (RA-SPRT) chart for incomplete cytoreduction (CCR 2/3 procedures). The x-axis represents the operation number. The y-axis represents a cumulative log-likelihood ratio value.

The LC for the completeness of cytoreduction was achieved after 30 procedures, exceeding the assumed null hypothesis −5.2 (minimum value according to literature −15.3%, maximum −27%). The percentage of complete cytoreductions increased at a constant rate. Incomplete cytoreduction CCR 2/3 was performed in 26 cases (12.8%). The LC for the completeness of cytoreduction based on the number of incomplete CCR 2/3 cytoreductions is shown in Figure 1. 

According to the Clavien–Dindo classification, after approximately 50 procedures the LC oscillated around the assumed hypothesis h0 = −8.9 (minimum and maximum value according to the literature: 16.6% and 22.9%, respectively). The LC decreased after 109 procedures performed when the assumed number of complications had been reached. Complications in grade III and IV according to the Clavien–Dindo classification occurred in 47 patients (23%).

**Figure 2:**
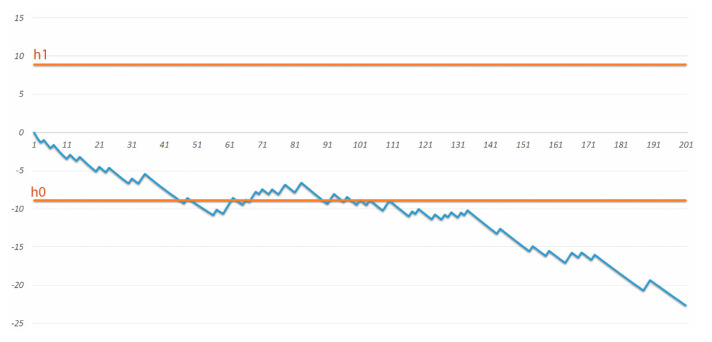
Risk-adjusted sequential probability ratio test (RA-SPRT) chart for complications according to the Clavien–Dindo classification. The x-axis represents the operation number. The y-axis represents a cumulative log-likelihood ratio value.

Until the first 109 CRS + HIPEC procedures, the complication rate was 28% with a decrease to 20% in subsequent patients. The difference was statistically significant (p = 0.05). The LC RA-SPRT chart for complications according to the Clavien–Dindo classification is shown in Figure 2.

**Figure 3:**
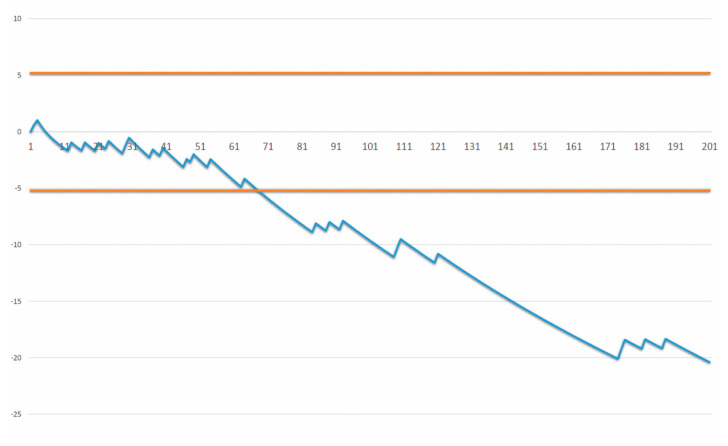
Risk-adjusted sequential probability ratio test (RA-SPRT) chart for complications according to comprehensive complication index (cut off value = 40). The x-axis represents the operation number. The y-axis represents a cumulative log-likelihood ratio value.

CCI complication rate and length of stay (LOS) were plotted in a graph using the moving-average (MA) method (Figure 3.).

**Figure 4:**
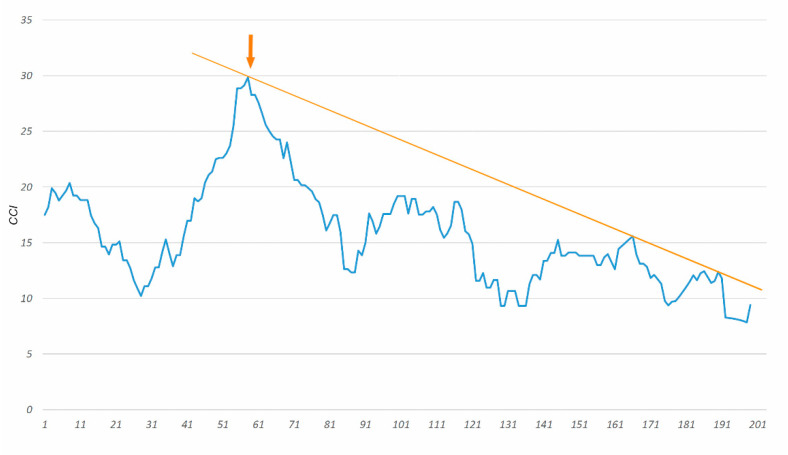
Moving average of estimated risk of complications according to comprehensive complication index (cut off 40). The x-axis represents the operation number.

The LC according to the comprehensive complication index is illustrated in Figure 4. After 56 procedures, the moving average reached a maximum of 30, and after evaluating subsequent cases, the curve oscillated from 8 to 20 with a clear downward trend.

**Figure 5:**
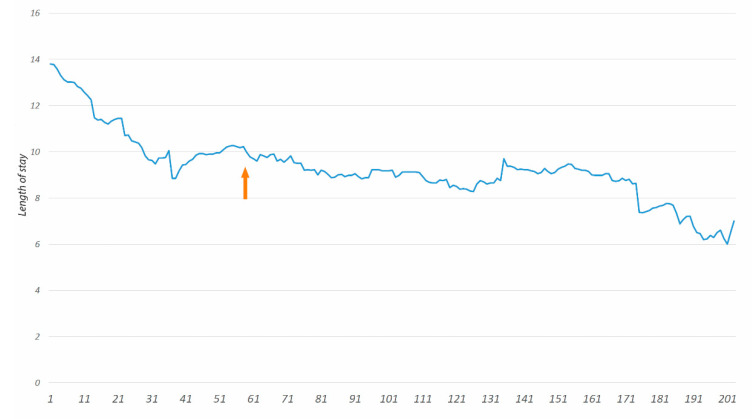
Moving average plot for length of stay (LOS). The x-axis represents the operation number. In all subsequent cases after arrow, the moving average (MA) of LOS is below 10 days.

The LC according to the length of stay (LOS) is shown in Figure 5. After 58 CRS + HIPEC procedures performed, the LC tends to decrease. The MA of LOS in subsequent cases is below 10.

The number of CRS + HIPEC procedures necessary to reach a *plateau* for the LC for various oncological (completeness of cytoreduction) and surgical factors (LOS, complications according to CCI and Clavien–Dindo classification) is shown in Table 5.

## 3. Discussion

This study used SPRT to determine the LC for CRS + HIPEC and assess the minimum number of performed procedures to achieve the necessary experience in the treatment of PSM. Our single institution 8 years (2010–2018) of experience of 203 CRS and HIPEC procedures in 185 patients with PSM has demonstrated acceptable perioperative outcome and long-term survival. Complete cytoreduction (CCR 0/1) was achieved in the majority (88%) of patients. The postoperative mortality (30- and 90-days) rate was 2% and 4%, respectively. Severe (grade III/IV) complications according to Clavien–Dindo classification occurred in 23% of patients. The median CCI was 21 (mean 31). The median LOS was 8 days (mean ± SD; range: 10.5 ± 8.0; 4–57). The number of operations required to achieve the plateau of the LC for complete cytoreduction and complications according to the Clavien–Dindo classification or the CCI, were 30, 109, or 56, respectively. The LOS reached the plateau after 58 operations. 

The CRS followed by HIPEC represents a treatment option for PSM. Although improved survival has been reported in selected patients, it is still associated with high morbidity and mortality. The latter two, as well as complete cytoreduction, have been commonly used to assess the LC to show acceptable outcomes of the CRS and HIPEC. The reduction of postoperative complications after CRS and HIPEC is essential for optimal short- and long-term outcomes. For assessing the total burden of postoperative complications per patient, the CCI has been reported to be superior to the Clavien–Dindo classification and more sensitive for assessing surgery- and cancer-related outcomes [29]. The monitoring of these outcomes is essential for the introduction of the CRS and HIPEC in a single institution and it enables to verify the effectiveness of the morbid procedure. Moreover, severe postoperative complications have a major influence on costs after CRS and HIPEC and result in a threefold increase in hospital costs in affected patients [30]. 

Over 20 years (1995–2017) single Italian center experience and analysis of 300 patients who underwent CRS and HIPEC revealed a morbidity rate of 36% [31]. According to the Clavien–Dindo classification, 22% were grade I–II complications and 12% were grade III–IV. Reoperation was needed in 9% of patients. The mortality rate was 2.3%. Similar early results, as well as favorable survival outcome, can be achieved already after 6 years (2006–2012) in 80 selected patients if the CRS and HIPEC is centralized as in Denmark [32]. Short-term and long-term outcomes were comparable to results from international centers. Outcomes from a single tertiary US institution, based on 170 consecutive patients over a period of 5 years (2007–2012), revealed that the majority (77%) of patients could have complete cytoreduction (CC0–1) [33]. In this institution, in agreement with US guidelines [34], Mitomycin C (MMC) was administered intraperitoneally at 42°C for 90 minutes in the majority (89%) of patients. In the study of 474 consecutive patients treated with CRS and HIPEC over 5 years (2011–2015) in a high-volume certified PSM reference German center, the re-operation rate was 15% [35]. 

Voron et al., after the analysis of 290 consecutive CRS with HIPEC procedures in 204 patients, showed that 140 and 40 cases were needed to achieve the lowest risk of incomplete cytoreduction and major morbidity, respectively [14]. It has been suggested that, at the beginning of the LC, it is better to perform CRS and HIPEC in patients with colorectal, appendiceal, and ovarian cancer, avoiding those with pseudomyxoma peritonei and peritoneal mesothelioma [14]. Kusamura et al. demonstrated that 137 and 149 cases were needed to achieve appropriate complete cytoreduction skills and an acceptable severe complication rate, respectively. They suggested that approximately 140 cases were needed to achieve proficiency in CRS and HIPEC procedure [24]. Australian authors, after a retrospective revision of 800 consecutive patients treated by CRS and HIPEC, reported 200 cases needed to achieve appropriate complete cytoreduction and major morbidity rates. Moreover, the rate of required blood transfusions was significantly lower after 100 performed procedures [16]. Polanco et.al. showed that approximately 190 cases are needed to achieve completeness of cytoreduction and the lowest risk of severe morbidity and mortality [36]. Evaluation of LC in the complex treatment of PSM by Clavien–Dindo Classification/CCI, as shown in our study, provides new insight into the postoperative outcome of patients undergoing CRS + HIPEC. At the time of study published by Kusamura et al [24] in 2012, the CCI was not known since it was introduced a year later by Slankamenac et al [27]. Moreover, information gained by the CCI increases with the complexity of the surgery, such as multivisceral resections done for complete cytoreduction with HIPEC [27].

Several methods of delivering HIPEC have been described, but no significant differences in treatment outcomes, morbidity, or safety have been found among them, and the ultimate choice between them is left to individual preference or institutional criteria [37]. We may only speculate whether an initially used (first 107 procedures) open (coliseum) technique, followed by a closed HIPEC technique, as well as intraperitoneal monotherapy, MMC, followed by oxaliplatin in moderate doses (30 and 300 mg per m^2^, respectively), contributed to the low morbidity rate. Mentoring is a key factor to shorten the LC and ensure the quality of the training in CRS and HIPEC [17]. When introduced without a mentorship program, the LC for CRS + HIPEC will be shorter in patients with low PCI when compared to patients with severe peritoneal dissemination. The present series supports the safety of CRS and HIPEC in selected patients with PSM, even in a setting where no established mentorship program had been introduced.

## 4. Materials and Methods

After having institutional review board approval (Ethics code: KE-0254/297/2018), data were analyzed from a prospectively maintained database. Between November 2010 and August 2018, 300 patients with primary or secondary malignancies of the peritoneal surface were qualified for the multimodal treatment. Each time patients were assessed by a multidisciplinary team, which included medical oncologists, surgical oncologists, radiation oncologists, anesthesiologists, pathologists, radiologists, and psychologists. The inclusion criteria included: primary and secondary PSM with limited carcinomatosis, grade ≤ III of the American Society of Anesthesiologists (ASA) physical status classification system, grade ≤ III according to Eastern Cooperative Oncology Group (ECOG) classification. The exclusion criteria included: the lack of the possibility to perform efficient cytoreductive surgery considering severe carcinomatosis, clinically apparent nodal recurrence for secondary malignancies or an inability to confirm the presence of the disease. All procedures were performed by two experienced surgeons (WP, JM). A total of 218 CRS and HIPEC procedures were ultimately performed in 185 patients.

For the objective evaluation of the distribution and volume of PM, a Sugarbaker’s peritoneal cancer index (PCI) was used [38]. It is calculated and given as a score ranging from 0 to 39 points, which describes the size of all the lesions throughout the peritoneal cavity divided into 13 regions. Resection of PM was performed according to previously described principles to clear the entire malignant peritoneal surface [39,40]. The CCR is defined in the 4-point scale: 0—complete removal of all the tumor tissues; 1—remaining tumor foci do not exceed 2.5 mm (total cytoreduction, full penetration of cytotoxic drugs into tumor tissue); 2—lesions in size from 2.5 mm to 2.5 cm—incomplete cytoreduction (moderate residual disease); 3—remaining lesions larger than 2.5 cm (macroscopic residual disease) [40]. Extensive CRS was considered when at least 3 organs were resected or at least 2 anastomoses were performed [41]. The prior surgical score (PSS) was used to assess the extent of the previous surgery [42]: PSS 0 indicated a diagnosis of PSM through biopsy alone, PSS 1 indicated exploration and surgery in 1 abdominal region, PSS 2 indicated exploration and surgery in 2–5 abdominal regions, whereas PSS 3 indicated exploration and surgery in over 5 abdominal regions.

After CRS completion, the patients underwent HIPEC. Intraperitoneal perfusions were performed using SunChip (Gamidatech^®^, Eaubonne, France). From November 2010 to December 2015, HIPEC was performed as an open procedure (Coliseum technique, Lublin, Poland), with a usage of the Münster retractor. Since December 2015, the HIPEC procedure was performed as a closed method. Two monotherapy protocols were used, with either 30 mg Mitomycin C dissolved in 0.9% NaCl at 42 °C for 60 min, or 300 mg/m^2^ Oxaliplatin dissolved in 5% glucose at 43 °C for 30 min, with a drug flow of 4–11 liters/minute. Throughout the procedure, the transoesophageal body temperature was additionally monitored. Critical hyperthermia was not reported. For the assessment of surgical complications, the Clavien–Dindo classification [28,43] and the CCI classification [44,45] were used. CCI is based on the widely established Clavien–Dindo classification, integrating all complications, including their severity on a linear scale ranging from 0 (no complication) to 100 (death) [27]. Grades I and II were considered minor complications, and grades III–V were considered major complications. For the latter one, a literature-based cut-off point of 40 points was adopted for further statistical assessment [46].

### Statistical Analysis

SPRT offers controlling the statistical process by enabling formal testing of the hypothesis. In clinical practice, SPRT allows for monitoring the safety of medical interventions [21]. In comparison to other methods of statistical process control, the benefit of SPRT lies in the fact that it enables us to carry out formal hypothesis testing. Due to the availability of functions for selecting type I and II error rates and the threshold of an unacceptable odds ratio (OR) for the result, the SPRT can be used to determine whether the available information is sufficient, or whether the hypothesis has been accepted or rejected. In addition, SPRT provides a graphic summary of changes in performance, which can reveal suboptimal performance. In SPRT analysis, four parameters were defined: predicted OR for complications according to the Clavien–Dindo III–V classification, incomplete CCR 2/3 cytoreduction, and I- and II-type error rates. The probability of both I- and II-type errors were set at 0.05. The two boundaries (hypothesis h0 and h1) and the cumulative sum of the log-likelihood ratio were calculated according to the equations shown in Table 6 [24,25]. OR was set to 1.4 to detect an unacceptable increase in the complication rate according to the Clavien–Dindo III–V classification and was estimated based on literature data. Following the study conducted by Kusamura et al. [24], the complication rates were set at 16.6% [47] and 22.9% [48] for the lower and upper thresholds, respectively. The obtained ratio between these two values (22.9/16.6) was approximately 1.4. In the assessment of cytoreduction, the OR value was set at 1.8. Incomplete cytoreduction rates were taken from the results of a multicentre study by Glehen et al. (15.3%) and Elias et al. (27%) [18,49]. The obtained ratio between these two values (27,0/15,3) was approximately 1.8.

The minimum number of operated cases necessary to determine the LC in CRS + HIPEC was estimated as the point at which the cumulative sum of the log reliability index exceeded the lower control limit (h0). H0 was set by the RA-SPRT model for the Clavien–Dindo III–V complication rate and incomplete CCR 2/3 cytoreduction rate. The LC was also assessed due to LOS, CCI complication rate by using the moving average method (MA) with a smoothing constant of 30 [50]. Overall survival (OS) was defined as the length of time from the date of CRS + HIPEC to the patient’s death or last documented follow-up.

The level of statistical significance was set at p ≤ 0.05. The simulated data were processed using MedCalc 19.1 (MedCalc Software, Ostend, Belgium). The LC and MA were calculated in Excel Microsoft Office 2013.

## 5. Conclusions

Some 110 procedures should be performed to achieve reproducible complete cytoreduction and an acceptable rate of severe complications and postoperative mortality. The risks of perioperative morbidity and mortality after CRS and HIPEC are analogous to any other major gastrointestinal surgery. CRS and HIPEC should remain a treatment option for highly selected patients in whom curative or life-prolonging treatment is a pursuit.

## Figures and Tables

**Table 1:** Demographic data and clinical features of patients treated with CRS + HIPEC.

Characteristic	Values
Number of patients	185
Mean age (year) ± SD; median, range	54 ± 10,3; 55, 20–75
Female/male	143/42
Number of patients with HIPEC procedure × 2 n (%)	10 (5,4%)
Number of patients with HIPEC procedure × 3 n (%)	4 (2,2%)
Number of HIPEC performed	203
Tumor histotype n (%)	
Ovarian	57 (31%)
Colorectal	41 (22%)
Gastric	30 (16%)
Pseudomyxoma peritonei	27 (15%)
Appendiceal	11 (6%)
Peritoneal mesothelioma	10 (5%)
Other types of peritoneal surface malignancy*	9 (5%)
ASA**	
II	48(26%)
III	137(74%)
ECOG	
I	53 (29%)
II	96 (52%)
III	36 (19%)
Preoperative chemotherapy n (%)	126(68%)
Prior surgical score	
0 (Biopsy alone)	16 (8%)
1 (Exploration and surgery in 1 region)	37 (18%)
2 (Exploration and surgery in 2–5 regions)	102 (50%)
3 (Exploration and surgery in >5 regions)	48 (24%)
Relapse of the disease n (%)	72 (39%)

* primary peritoneal cancer, small intestine cancer, mucous pancreatic cancer, cholangiocarcinoma. ** American Society of Anesthesiologists Class.

**Table 2:** Characteristics of cytoreductive surgery (CRS) and hyperthermic intraperitoneal chemotherapy (HIPEC) procedures.

Characteristic	Values
Mean cytoreductive surgery time (min) ± SD; median, range	179 ± 68, 4; 180, 60–420
HIPEC drug (application time and temperature) n (%)	
Oxaliplatin (30 min 43 °C)	116 (57%)
Mitomycin (60 min 42 °C)	87 (43%)
HIPEC technique n (%)	
Open	107 (53%)
Closed	93 (46%)
Laparoscopy	3(1%)
Mean peritoneal cancer index ± SD; median, range	10, 4 ± 8,0; 9, 0–34
Completeness of cytoreduction score n (%)	
CCR0	130 (64%)
CCR1	47 (24%)
CCR2	23 (11%)
CCR3	3 (1%)

**Table 3:** Spectrum of CRS + HIPEC procedures performed in the study.

Procedures	No. of Patients n (%)
**Peritonectomy**	218 (100%)
Right subdiaphragmatic Left subdiaphragmatic	56 (26%) 22 (10%)
Right lateral Left lateral Pelvic	65 (30%) 65 (30%) 88 (40%)
**Greater omentectomy**	109 (50%)
**Splenectomy**	27 (12%)
**Lesser omentectomy**	57 (26%)
**Segmental intestinal resection**	44 (20%)
**Colectomy**	56 (26%)
**Low anterior resection**	27 (12%)
**Distal Pancreatectomy**	6 (3%)
**Liver resection** Metastasectomy Segmentectomy Bisegmentectomy	25 (11%) 15 (9%) 5 (3%) 5 (3%)
**Appendectomy**	59 (27%)
**Gastrectomy** Total Subtotal	25 (11%) 22 (10%) 3 (1%)
**Oophorectomy**	6 (3%)
**Cholecystectomy**	24 (11%)
**Removal of single peritoneal metastases**	168 (77%)
PCI region: 1 2 3 4 5 6 7 8 0	25(11%) 15 (9%) 12 (8%) 30 (13%) 20 (10%) 18 (9%) 22 (10%) 16 (9%) 10 (6%)

**Table 4:** Postoperative complications for CRS + HIPEC according to Clavien–Dindo classification.

Characteristic	Values
Clavien–Dindo classification grade n (%)	
None	86 (42%)
I	24 (12%)
II	42 (21%)
III	15 (7%)
IV	32 (16%)
V (30 days)	4 (2%)
Tumor histotype n (%) Ovarian Colorectal Gastric Pseudomyxoma peritonei Appendiceal Peritoneal mesothelioma Cholangiocarcinoma	13/57 (23%) 9/41 (22%) 14/30 (47%) 5/27 (18%) 3/11 (27%) 3/10 (30%) 1/9 (11%)
Procedure-related mortality (30/90 days) n (%)	4/8 (2/4%)
In-hospital severe morbidity (III and IV grade) n (%)	47 (23%)
Re-operation	22 (11%)
Mean comprehensive complication index CCI ± SD; median, range	31,3 ± 21,2; 20,9 8,7–100
Comprehensive complication index CCI ≥ 40 n (%)	37 (18%)
Mean blood loss during surgery (mL) ± SD; median, range	425 ± 341; 500, 0–1800
Blood transfusions n (%)	43 (21%)
Mean number of blood units transfused (200 ml) ± SD; median, range	1,2 ± 2,1; 0, 0–12
Number of patients requiring stay in ICUs n (%)	33 (16%)
Mean ICU LOS (days) ± SD; median, range	3,75 ± 2,4; 4, 1–14
Mean hospital LOS (days) ± SD; median, range	10,5 ± 8,0; 8, 4–57

ICU, intensive care unit; LOS, length of stay.

**Table 5:** The number of required procedures necessary to accomplish the learning curve (LC) using various outcome parameters.

Variables	Number of Required Procedures (n)	Method
Completeness of cytoreduction	30	RA-SPRT
Complications qualitatively according to Clavien–Dindo classification	109	RA-SPRT
Complications according to CCI classification (cut off 40)	66	RA-SPRT
Complications quantitatively according to CCI classification	56	MA
LOS	58	MA

RA-SPRT, risk-adjusted sequential probability ratio test; MA, moving average; LOS, length of stay.

**Table 6:** Equations and variables to construct a risk-adjusted sequential probability test (RA-SPRT) chart [24,25].

Variable	Calculations	CCR 2/3 Cytoreduction	Clavien–Dindo III–V Complications Rate
OR₀	Odds ratio of acceptable outcome	1	1
OR₁	Odds ratio of unacceptable outcome	1.8	1.4
α	Probability type I error	0.05	0.05
β	Probability type II error	0.05	0.05
h₀	−ln [(1−*α*)/*β*]/ln (OR_1_)	−5.2	−8.9
h₁	ln [(1−*β*)/*α*]/ln (OR_1_)	5.2	8.9
T^cum^_i_	T^cum^_i−1_ + (O_i_ − *s*_i_)	Variable according to *P*_i_	Variable according to *P*_i_
*s*_i_	ln [(−*P*_i_) + (OR_1_ × *P*_i_)]/ln (OR_1_)		
Oi	Observed outcome for *i*th case	0 or 1	0 or 1
*P*i	Estimated probability of the outcome for the *i*th case as determined by the risk prediction model.	Variable according to the case	Variable according to the case
